# *SVP*-like MADS Box Genes Control Dormancy and Budbreak in Apple

**DOI:** 10.3389/fpls.2017.00477

**Published:** 2017-04-04

**Authors:** Rongmei Wu, Sumathi Tomes, Sakuntala Karunairetnam, Stuart D. Tustin, Roger P. Hellens, Andrew C. Allan, Richard C. Macknight, Erika Varkonyi-Gasic

**Affiliations:** ^1^The New Zealand Institute for Plant & Food Research Limited – Plant & Food ResearchAuckland, New Zealand; ^2^The New Zealand Institute for Plant & Food Research Limited – Plant & Food ResearchHawke’s Bay, New Zealand; ^3^School of Biological Sciences, University of AucklandAuckland, New Zealand; ^4^Department of Biochemistry, University of OtagoDunedin, New Zealand

**Keywords:** apple, *DAM*, SVP, dormancy, ectopic expression, biennial bearing

## Abstract

The annual growth cycle of trees is the result of seasonal cues. The onset of winter triggers an endodormant state preventing bud growth and, once a chilling requirement is satisfied, these buds enter an ecodormant state and resume growing. MADS-box genes with similarity to *Arabidopsis SHORT VEGETATIVE PHASE* (*SVP*) [the *SVP*-like and *DORMANCY ASSOCIATED MADS-BOX* (*DAM*) genes] have been implicated in regulating flowering and growth-dormancy cycles in perennials. Here, we identified and characterized three *DAM*-like (*MdDAMs*) and two *SHORT VEGETATIVE PHASE*-like (*MdSVPs*) genes from apple (*Malus × domestica* ‘Royal Gala’). The expression of *MdDAMa* and *MdDAMc* indicated they may play a role in triggering autumn growth cessation. In contrast, the expression of *MdDAMb, MdSVPa and MdSVPb* suggested a role in maintaining bud dormancy. Consistent with this, ectopic expression of *MdDAMb* and *MdSVPa* in ‘Royal Gala’ apple plants resulted in delayed budbreak and architecture change due to constrained lateral shoot outgrowth, but normal flower and fruit development. The association of *MdSVPa* and *MdSVPb* expression with floral bud development in the low fruiting ‘Off’ trees of a biennial bearing cultivar ‘Sciros’ suggested the *SVP* genes might also play a role in floral meristem identity.

## Introduction

The annual growth cycle of trees in temperate zones consists of dormant and active growth phases. External environmental factors such as cold or drought stress trigger the transition to dormant phase, resulting in shoot growth cessation and bud set. Subsequently, internal signals in the bud are induced to prevent its growth during the endodormant state (Lang et al., 1987; Horvath et al., 2003). These buds shift to the ecodormant state after satisfying the chilling requirement, where buds are capable of resuming growth (Lang et al., 1987).

Recent studies in *Populus* revealed striking similarities between genes and pathways that regulate flowering time in annuals and growth and dormancy cycles in perennials, as reviewed by Ding and Nilsson (2016); homologs of circadian-clock and photoperiod pathway components phytochromes and *CONSTANS* (*CO*) impact on growth cessation, homologs of key flowering genes *FLOWERING LOCUS T* (*FT*), bZIP transcription factor *FD* and floral meristem identity gene *APETALA1* (*AP1*) have all been shown to regulate growth, while a homolog of *TERMINAL FLOWER1* (*TFL1*) affects chilling-requirement and dormancy release. A role of hormones, particularly abscisic acid (ABA) has been well established in both bud and seed dormancy (Rohde and Bhalerao, 2007; Horvath et al., 2008; Graeber et al., 2012) and commonalities in genes that regulate bud and seed dormancy were reported in peach (Leida et al., 2012a; Wang et al., 2016). A novel regulator, putative APETALA2/Ethylene responsive transcription factor *EARLY BUD-BREAK 1* (*EBB1*) was also identified as a determinant of the time of bud break in *Populus* (Yordanov et al., 2014) and subsequently associated with bud break regulation in apple (Wisniewski et al., 2015). Ectopic expression of a peach C-repeat binding factor (CBF/DREB) gene *PpCBF1* in apple resulted in unexpected short-day induced dormancy and delayed budbreak in spring (Wisniewski et al., 2011; Artlip et al., 2014), demonstrating a role of a cold-tolerance gene in regulation of growth and dormancy in trees.

Another class of genes clearly associated with regulation of flowering in annuals and dormancy in perennials are MADS-box genes with similarity to *Arabidopsis SHORT VEGETATIVE PHASE* (*SVP*). Deletion of six *DORMANCY ASSOCIATED MADS-BOX* (*DAM*) genes in the *EVERGROWING (EVG)* locus resulted in a complete lack of dormancy in the peach (*Prunus persica*) *evergrowing* (*evg*) mutant (Bielenberg et al., 2004, 2008). Peach *DAM* gene expression tracks seasonal light and temperature cycles, integrating environmental cues that regulate the transition into and out of endodormancy (Li et al., 2009). *DAM1, DAM 2* and *DAM 4* showed elevated expression over summer and early autumn, which coincided with the timing of short-day induced growth cessation. Therefore, these three *DAM* genes were inferred as the most likely candidates for the cause of the non-dormant bud set phenotype seen in the *evg* mutant trees (Li et al., 2009). *DAM3* did not show any distinct expression pattern throughout the annual cycle, but *DAM5* and *DAM6* expression was elevated over the winter, suggesting that they may play a role in the maintenance of bud endodormancy (Li et al., 2009). Correspondingly, overexpression of the Japanese apricot (*Prunus mume*) homolog of *DAM6* in poplar caused early growth cessation and early terminal bud set, confirming the growth inhibitory function (Sasaki et al., 2011). The growth inhibitory function was also confirmed in kiwifruit. Overexpression of *Actinidia chinensis SVP3* retarded reproductive development in transgenic *A. eriantha* (Wu et al., 2014) and overexpression of *SVP2* delayed budbreak in transgenic *A. deliciosa* (Wu et al., 2017).

In Rosaceae, *DAM* genes form a subclade separate from true *Arabidopsis SVP*-like and potato *StMADS11*-like subclades (Velasco et al., 2010; Wells et al., 2015; Niu et al., 2016; Porto et al., 2016). In peach, the *SVP* gene family is expanded to six *DAM* genes, tandemly arranged on peach chromosome 1 (Jimenez et al., 2009; Illa et al., 2011), one true *SVP* ortholog *PpeMADS57* and the less similar *PpeMADS58*, located on chromosomes 6 and 8, respectively (Wells et al., 2015). A similar expansion of six tandemly arranged *DAM* genes was found on Japanese apricot chromosome 2, but two *SVP* orthologs are located on chromosomes 1 and 6 (Sasaki et al., 2011; Xu et al., 2014). In pear, three *DAM* and one *SVP* orthologs have been identified (Ubi et al., 2010; Niu et al., 2016). Several studies attempted to identify and characterize apple *DAM* genes after the apple genome sequence release (Velasco et al., 2010). The presence of *DAM* and *SVP* orthologs was confirmed (Mimida et al., 2015; Wisniewski et al., 2015; Kumar G. et al., 2016; Porto et al., 2016), with some discrepancies in nomenclature used in reports and the possible presence of misannotated genes or pseudogenes (Mimida et al., 2015; Porto et al., 2016). Despite the nomenclature discrepancies, apple *DAM* genes appear to be arranged in tandem on chromosomes 8 and 16, in regions syntenic to the peach chromosome 1, where six peach *DAM* genes are located (Illa et al., 2011; Mimida et al., 2015; Porto et al., 2016). Two *SVP* orthologs are located on apple chromosomes 4 and 11, respectively. Ectopic expression of the *SVP* ortholog *MdJOINTLESSa* (*MdJa*) restored the *JOINTLESS*-deficient tomato mutant (Nakano et al., 2015), demonstrating similar molecular activity between *SVP*-like genes, while differential seasonal expression of apple *DAM* and *SVP* genes at different stages of apical bud development suggested roles in bud dormancy (da Silveira Falavigna et al., 2013; Mimida et al., 2015; Porto et al., 2016). Seasonality and differential expression patterns were also demonstrated in bark tissue of apple (Wisniewski et al., 2015). In addition, sharp spikes in expression in bark and vegetative buds of a transgenic *PpCBF1* apple line with altered cold acclimation, dormancy, and growth suggested for distinct roles in regulation of bud dormancy and potentially cambial dormancy (Wisniewski et al., 2015).

So far there has been no attempt at functional characterization of apple *DAM* and *SVP* genes by transgenic overexpression in apple. Similarly, little is known about the role of these genes in floral induction, flower and fruit development and if they contribute to biennial bearing, i.e., limited flowering after a year with heavy fruit load, which has been a major constraint on fruit production in many apple cultivars.

In this study, *DAM* and *SVP* homologs were identified and tested for expression in apple tissues and in apical buds during the growth and dormancy cycle. Potential roles in regulation of flowering were examined using apical buds of fruit-bearing and non-bearing trees. To examine whether apple *DAM* and *SVP* genes play the same or similar roles in apple, a full-length apple *DAM* cDNA (*MdDAMb*) and a full-length *SVP* cDNA (*MdSVPa*) were characterized using ectopic overexpression in transgenic apple. Their role in growth retardation is discussed.

## Materials and Methods

### Apple Tissue Sample Collections

Apple (*Malus × domestica* ‘Royal Gala’) samples were collected from 3-year-old plants grown in a glasshouse at ambient conditions. Three plants were used for sampling. Root, stem, leaf, flower and fruit tissue sampling was performed during the spring to autumn season of 2008 in a glasshouse at Plant & Food Research, Mt Albert, Auckland, New Zealand. Flowers were collected 5 days after bloom (October 2008) and fruit 132 days after bloom. The remaining tissues were sampled at the beginning of summer (December 2008), except for apical buds which were sampled in early autumn (March 2008). Apical bud samples were collected from fruit bearing and non-fruit bearing ‘Sciros’/Pacific Rose^TM^ apple trees grown in the field under standard orchard management. In total, 10 fruit bearing and 10 non-fruit bearing plants were used. Because of the bud tissue availability and the duration of the experiment, buds from multiple trees had to be used as one biological replicate and two replicates were collected at each sampling date. The sampling was performed at approximately four-weekly intervals from early summer 2011 to late spring 2012 at the Plant & Food Research orchard near Havelock North, New Zealand. The beginning of leaf drop was the last week of May 2012, 50% of budbreak was recorded at the second week of September 2012 and 80% full-bloom in the first week of October 2012.

### Identification and Phylogenetic Study of ‘Royal Gala’ *SVP* and *DAM*-like Genes

The full-length apple ‘Royal Gala’ *DAM*-like (*MdDAMb*) and *SVP*-like (*MdSVPa*) transcripts were identified from the Plant & Food Research *Malus* EST database (Newcomb et al., 2006) by BLAST alignment (Altschul et al., 1990). The remaining *DAM*-like and *SVP*-like coding sequences were amplified using oligonucleotide primers designed from ‘Royal Gala’ partial sequences and previously reported apple *DAM*/*SVP*/*JOINTLESS* genes (Supplementary Table S1). The predicted full length amino acid sequences were aligned with the full length of amino acid sequences of *Arabidopsis SVP* (AT2G22450) and *AGL24* (AT4G24540), potato *StMADS11* (AF008652), apple *MdDAM1* (KT582786), *MdDAM2* (KT582787), *MdDAM4* (KT582789), *MdJb* (LC004730), apricot *PmDAM6* (AB437345), leafy spurge *EeDAM2* (EU339320), three pear *DAM* (*PpyDAM1*, *PpyDAM2* and *PpyDAM3*) and one pear *SVP* (*PpySVP)* (Niu et al., 2016), six peach *DAM* (*PpeDAM1, PpeDAM2, PpeDAM3, PpeDAM4, PpeDAM5* and *PpeDAM6)* (Bielenberg et al., 2006) and one peach *SVP* (*PpeMADS57*) (Wells et al., 2015), trifoliate orange *PtSVP* (FJ373211), kiwifruit *SVP* gene*s* (*AcSVP1*, *AcSVP2, AcSVP3* and *AcSVP4*) (Wu et al., 2012). The alignments were performed using Clustal W (opening = 10, extension = 0.2). Phylogenetic and molecular evolutionary analyses were conducted using MEGA version 7.0 (Kumar S. et al., 2016), minimum evolution phylogeny tested with 1000 bootstrap replicates and above 50% of the cut-off value for condensed tree.

### RNA Extraction and Quantitative RT-PCR (qRT-PCR) Analysis

Apple bud and tissue RNA was extracted using the Spectrum Plant Total RNA kit (Sigma–Aldrich) according to the manufacturer’s instructions. Total RNA (5 μg) was treated with DNaseI (Ambion) and reverse-transcribed at 37°C using the BluePrint^®^ Reagent kit for RT-PCR (TaKaRa). Amplification and quantification were carried out using the LightCycler^®^ 480 System, LightCycler 480 SYBR Green I Master Mix and LightCycler 480 software version 1.5 (Roche Diagnostics). qPCR conditions were 5 min at 95°C, followed by 40 cycles of 5 s at 95°C, 5 s at 60°C, and 10 s at 72°C, followed by 65–95°C melting curve detection. Relative abundance was calculated using the ΔΔCT method and efficiency was corrected based on standard curves generated from cDNA serial dilutions. Oligonucleotide primers used in this study (Supplementary Table S1) were designed to amplify products between 150–300 bp in size. The reference gene *MdSGL29F* (GenBank accession number CN892118) was chosen because of demonstrated low-variance across various tissues, development and a range of treatments (Bowen et al., 2014). Other reference genes were considered and demonstrated similar patterns across the samples (**Supplementary Figure S1**). Three biological replicates (individual trees) were used for tissue-specific expression experiments. Two biological replicates were used for seasonal bud expression experiments. Each single transgenic plant (single line) was used to evaluate transgene expression levels; young leaf tissue was used for this experiment. Four technical replicates were used for each biological replicate.

### Plasmid Preparation and Plant Transformation

Full length cDNA sequences of *MdSVPa* and *MdDAMb* were cloned into a binary vector pART277 carrying the *CaMV 35S* promoter, derived from the binary vector pART27 (Gleave, 1992). A construct of the *35S* promoter-driven *uidA* (GUS) gene was used as a control. *Agrobacterium tumefaciens* strain LBA4404 containing the above binary vectors were transformed into the leaf disks of *M. × domestica* ‘Royal Gala’ plants, following a previously reported method (Yao et al., 1995). Transgenic plants were transferred to soil and grown under light and temperature conditions replicating the ambient environment in a containment glasshouse at Plant & Food Research, Auckland, New Zealand. Transgenic *MdDAMb* and *MdSVPa* plants and controls were carefully monitored at all stages of plant development.

## Results

### Apple ‘Royal Gala’ *DAM* and *SVP* Genes

Interrogation of the apple EST database (Newcomb et al., 2006) identified a full-length ‘Royal Gala’ *SVP*-like and a full-length ‘Royal Gala’ *DAM*-like sequence, previously annotated as *MdMADS16* (GeneBank accession number HM122599) and *MdMADS20* (GeneBank accession number HM122604), respectively. The predicted amino acid sequence of ‘Royal Gala’ MdMADS16 is identical to the previously described ‘Fuji’ MdSVPa and corresponds to MdJa (Nakano et al., 2015) and gene model MDP0000233948 on chromosome 11. The predicted amino acid sequence of ‘Royal Gala’ MdMADS20 is identical to previously reported ‘Fuji’ MdDAMb (Mimida, GeneBank accession number ADL36743), and corresponds to gene model MDP0000255146 on chromosome 16 (Supplementary Table S2).

**FIGURE 1:**
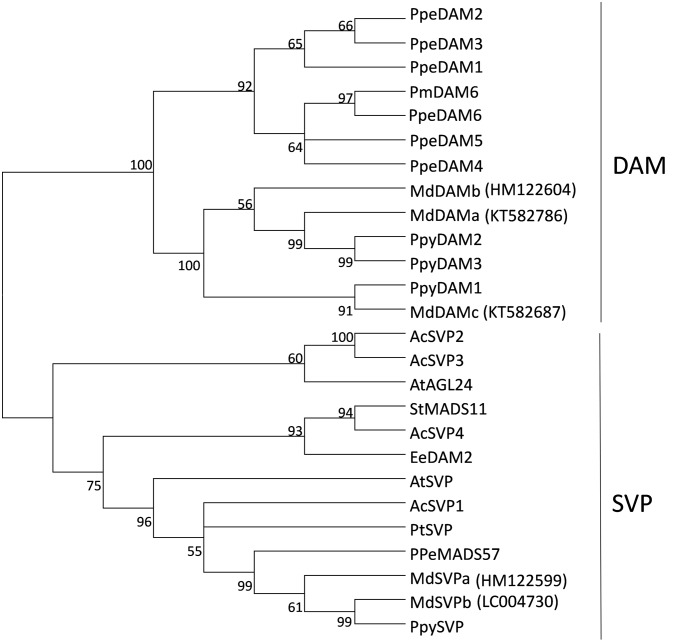
**Phylogenetic tree of DAM and SVP predicted proteins**. The tree was built from the full-length amino acid sequence alignment using the neighbor-joining method and the bootstrap test (1000 replicates). Bootstrap values above 50% are shown next to the branches. Ac, *Actinidia chinensis*, At, *Arabidopsis thaliana*, Ee, *Euphorbia esula*, Pm, *Prunus mume*, Ppe, *Prunus persica*, Ppy, *Pyrus pyrifolia*, Pt, *Poncirus trifoliata*, St, *Solanum tuberosum*, Md, *Malus × domestica* ‘Royal Gala’ sequences, with indicated accession numbers.

Further interrogation of ‘Royal Gala’ transcripts identified partial sequences that were highly similar to previously reported *MdDAMa*/*MdDAM1, MdDAMc*/*MdDAM2* and *MdSVPb*/*MdDAM3*/*MdJb* sequences (Mimida et al., 2015; Nakano et al., 2015; Wisniewski et al., 2015; Porto et al., 2016). Amplification of full-length sequences from ‘Royal Gala’ tissues confirmed that the ‘Royal Gala’ sequences were identical or almost identical to previous reports. However, we could not identify a ‘Royal Gala’ *MdDAMd*/*MdDAM3*, already suggested to be either a pseudogene or a result of misannotation (Mimida et al., 2015; Porto et al., 2016), nor *MdDAM4* (KT582789) (Porto et al., 2016). To prevent confusion, the nomenclature proposed by Mimida et al. (2015) was chosen (Supplementary Table S2) to reflect the relationship to *DAM* and true *SVP* homologs. Phylogenetic study of predicted full-length protein sequences confirmed the placement of ‘Royal Gala’ MdDAM and MdSVP proteins in the DAM and SVP subclades (**Figure 1**).

### Tissue-Specific Expression Profiles of *MdDAM* and *MdSVP* Genes

**FIGURE 2:**
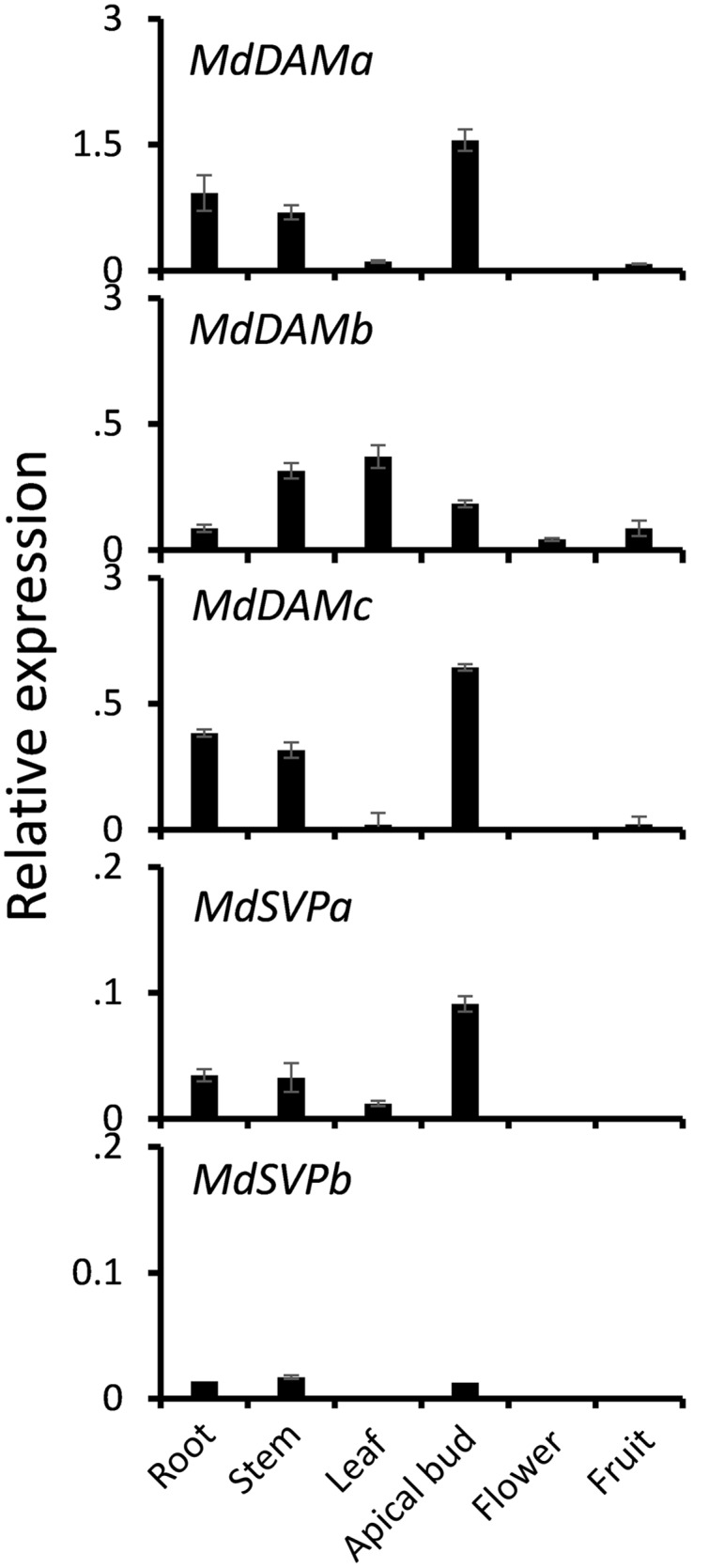
**Relative expression of *MdDAM* and *MdSVP* genes in root, stem, leaf, apical bud, flower, and fruit of apple**. The level of expression was normalized to apple reference gene, *MdSGL29F*. Error bars represent ± SE of three biological replicates.

To investigate whether *MdDAM* genes and *MdSVP* genes were expressed in a tissue-specific manner, qPCR was performed on extracts from various apple tissue samples. Transcripts of *MdDAM* and *MdSVP* were predominately found in vegetative tissues and apical buds (**Figure 2**). *MdDAMa* and *MdDAMc* were expressed in apical buds, root and stem. Transcripts of *MdDAMb* were predominantly found in leaf, stem, but also in root, flower and fruit. *MdSVPa* transcripts were detected in apical buds, root, stem and leaf tissues and a similar pattern but lower relative expression, particularly in apical buds, was detected for *MdSVPb*.

### Distinct Seasonal Expression Patterns in Apical Buds over a Growth and Dormancy Cycle

**FIGURE 3:**
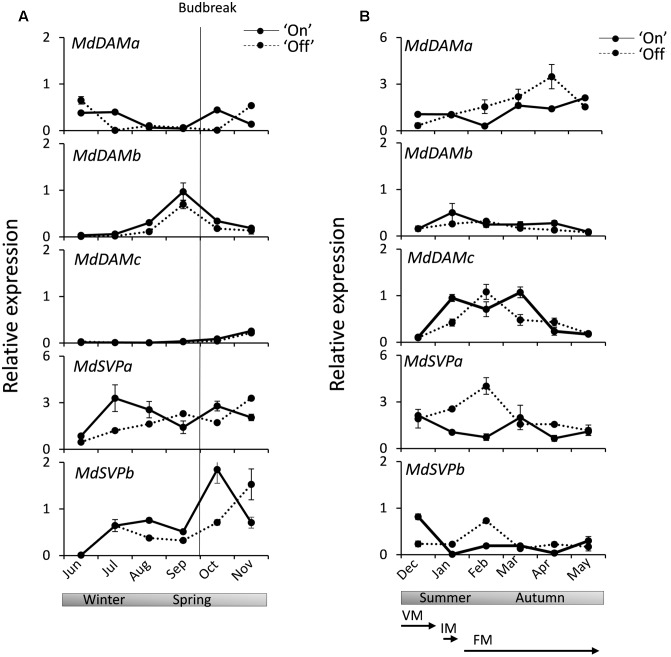
**Relative expression of *MdDAM* and *MdSVP* genes in apple apical buds during the season. (A)** *MdDAM* and *MdSVP* genes expression during winter dormancy and active growth in spring. **(B)** *MdDAM* and *MdSVP* genes expression from full bloom to leaf drop. The solid line represents samples collected from the fruit bearing (‘On’) trees. The dotted line represents samples collected from non-fruit bearing (‘Off’) trees. The level of expression was normalized to apple *MdSGL29F*. Data points represent the mean ± SE of two biological replicates. 50% budbreak is indicated by a vertical line. Stages representing predicted common terminal meristem identity in ‘Off’ trees are presented below. The timing of transitions between meristem identities was adopted from Foster et al. (2003): competent vegetative meristems (VM) transition to inflorescence meristems (IM) around 100 days after full bloom (DAFB) and to terminal floral meristem (FM) identity between 100 and 141 DAFB.

Expression analysis was performed for *MdSVP* and *MdDAM* genes in apical buds from ‘Sciros’ apple trees harvested over a full growing season, including autumn growth cessation and winter dormancy. This cultivar was chosen for its well-established alternating cropping pattern (Bertelsen and Tustin, 2002), with plants in the light and heavy flowering year of a biennial bearing cycle growing in the same orchard plot. Buds were harvested from fruit-bearing (‘On’) and non-bearing (‘Off’) trees to ascertain potential roles of these genes in flowering. Distinct patterns were observed in buds monitored between establishment of dormancy to reactivation of growth in spring (**Figure 3A**) and during the full bloom and fruit development stage to leaf drop, which coincide with transitions between meristem identities and subsequent floral organ development in ‘Off’ trees (**Figure 3B**). Transcripts of *MdDAMa* and *MdDAMc* were mostly absent in winter and spring months. *MdDAMb* was elevated in late winter, peaking in early spring, before visible budbreak. *MdSVPa* transcript was elevated during dormancy in winter months. Similar mid-winter accumulation was observed for *MdSVPb* (**Figure 3A**). *MdDAMc* transcripts accumulated in summer months and declined in autumn, while *MdDAMa* accumulated in summer and peaked in autumn (**Figure 3B**). Small differences, but similar general trends, were detected in buds collected from ‘On’ and ‘Off’ trees for all *MdDAM* genes. Steady *MdDAMa* and *MdDAMc* accumulation with single prominent peaks in April and February, respectively, were detected in ‘Off’ trees, while expression remained elevated over a longer period in ‘On’ trees (**Figure 3B**). More prominent differences in accumulation between ‘On’ and ‘Off’ trees were detected for *MdSVP* genes. Summer accumulation, with a peak in February, was observed for *MdSVPa* and *MdSVPb* in ‘Off’ trees (**Figure 3B**) and a faster accumulation rate of *MdSVPa* in winter and *MdSVPb* in spring were detected in ‘On’ trees (**Figure 3A**).

### Ectopic Overexpression of *MdDAMb* and *MdSVPa* Affects Apple Plant Development

**FIGURE 4:**
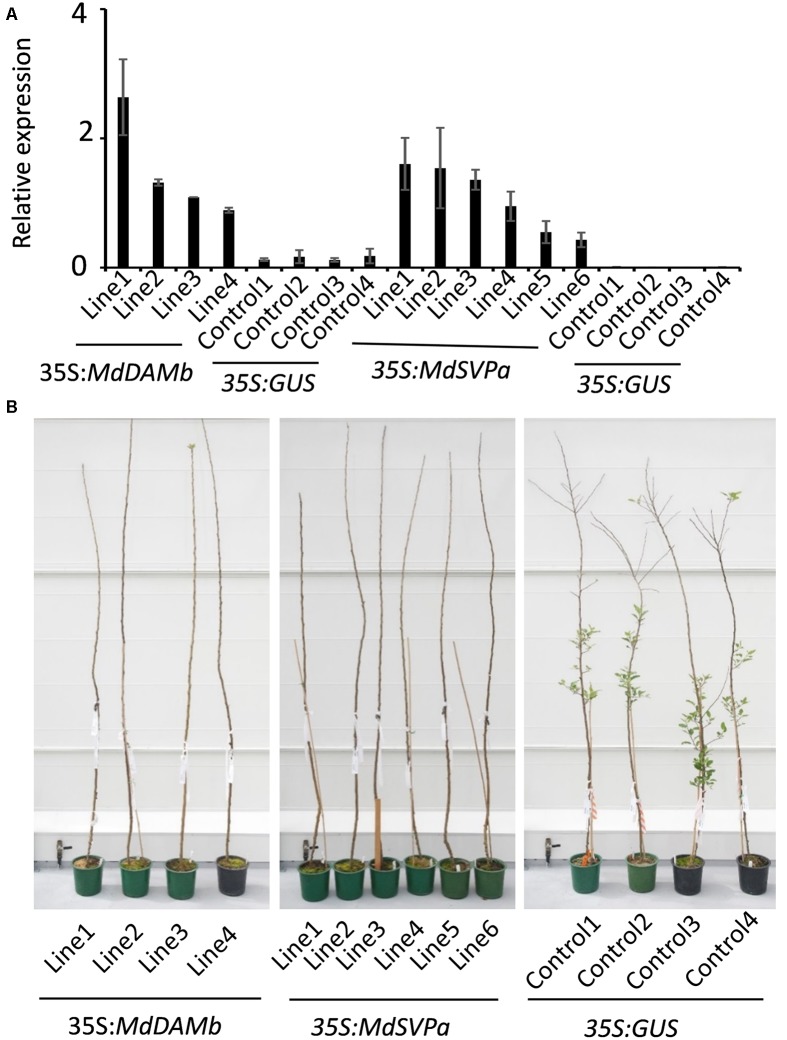
**Appearance of transgenic *MdDAMb* and *MdSVPa* ‘Royal Gala’ lines at the first year spring in the glasshouse. (A)** Relative expression of *MdDAMb* and *MdSVPa* in leaf tissue of transgenic ‘Royal Gala’ lines. The expression of each gene was normalized against apple *MdSGL29F*. Error bars represent SE for four technical replicates. **(B)** Phenotypes of constitutive expression of *MdDAMb* and *MdSVPa* in ‘Royal Gala’ compared with controls.

**FIGURE 5:**
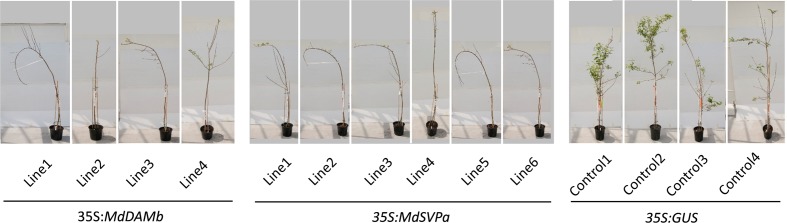
**Appearance of transgenic *MdDAMb* and *MdSVPa* ‘Royal Gala’ lines at the second year spring in the glasshouse**. All plants were pruned to induce lateral shoot growth.

**FIGURE 6:**
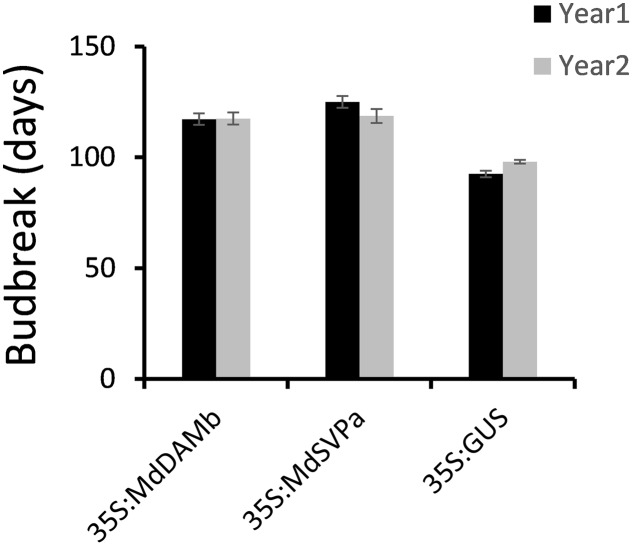
**Delayed budbreak in transgenic *MdDAMb* and *MdSVPa* ‘Royal Gala’ lines**. Budbreak was recorded as a number of days from 100% leaf drop in late autumn to the first visible leaf in spring and presented as the mean ± SE of individual transgenic lines.

For further functional characterization of apple *DAM* and *SVP* genes, ectopic overexpression of *MdDAMb* and *MdSVPa* was performed. Their full-length cDNAs driven by the CaMV *35S* promoter were transformed into ‘Royal Gala,’ using an established apple transformation protocol (Yao et al., 1995). Initially, a very low transformation efficiency and retarded growth were observed with both *35S:MdDAMb* and *35S:MdSVPa*, but not the control *35S:GUS* construct. After multiple transformation experiments, four *MdDAMb* and six *MdSVPa* transgenic lines were established (**Figure 4A**) and grown alongside four *35S:GUS* control lines for 2 years. Transgenic *MdDAMb* and *MdSVPa* plants had a strong apical dominance phenotype, with one main upright stem and constrained lateral shoot outgrowth during the first growing season (**Figure 4B**). Pruning of shoot tips and bending of the stems induced lateral shoot growth (**Figure 5**), but the timing of spring budbreak in transgenic *MdDAMb* and *MdSVPa* lines was significantly delayed in both years (**Figure 6**). No early leaf senescence and no early bud set could be detected in transgenic *MdDAMb* and *MdSVPa* lines over the 2 years of phenotypic analysis.

### Ectopic Overexpression of *MdDAMb* and *MdSVPa* Does Not Affect Apple Flower and Fruit Development

**FIGURE 7:**
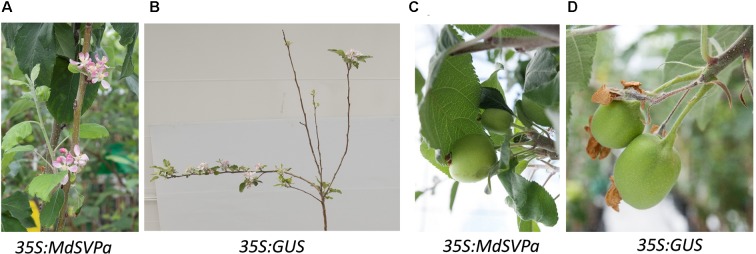
**Flowers and fruits on 35S:*MdSVPa* transgenic lines versus controls. (A)** The flowers of 35S:*MdSVPa* transgenic lines harbored on the main stem. **(B)** Typical position of flowers on control plants. **(C,D)** Fruit on 35S:*MdSVPa* transgenic lines and controls.

The first flowering was observed in the spring of the third year after transferring of plants into the glasshouse. The time of flowering (appearance of visible flower buds) was similar between transgenic *MdDAMb, MdSVPa* and control lines (data not shown). Flowers on less branched *MdSVPa* lines were mostly harbored on the main stem, in contrast to control plants where most flowers were found on lateral branches (**Figures 7A,B**), but the duration of flower and fruit development, as well as flower and fruit morphology appeared unaffected (**Figures 7C,D**).

## Discussion

In temperate horticultural trees, adequate timing of the onset and duration of winter dormancy are essential to avoid unfavorable winter conditions and ensure flowering in the following season. Despite the importance of dormancy in apple production, very little is known about the molecular processes underlying seasonal growth cycles. MADS-box genes with similarity to *SVP* were advanced as potential regulators for apple dormancy control (Mimida et al., 2015; Wisniewski et al., 2015; Porto et al., 2016), but the functional characterization using transgenic plants has not yet been reported. The objective of the present study was to functionally characterize apple *DAM* and *SVP* genes in transgenic apple ‘Royal Gala’ and perform expression analyses which would shed light on potential roles during apple vegetative and flower development.

Expression of three *DAM* and two *SVP* genes have been confirmed in tissues of apple cultivar ‘Royal Gala’ and further tested in apical buds of a biennial bearing apple cultivar ‘Sciros.’ The predominantly vegetative expression and distinct seasonal profiles suggested conserved roles in vegetative growth, but divergent functions in apple bud development and growth-dormancy cycles. The progressive upregulation of *MdDAMa* and *MdDAMc* during summer and autumn coincided with cessation of terminal growth, and displayed a pattern whereby *MdDAMc* accumulation peaks and declines preceded those of *MdDAMa.* This is highly similar to the pattern reported for other apple cultivars (Mimida et al., 2015; Wisniewski et al., 2015). These seasonal expression profiles concurred with patterns of peach *DAM1, DAM 2* and *DAM 4*, predicted to be the most likely candidates causing the non-dormant bud phenotype in the *evg* mutant (Li et al., 2009). On the other hand, expression of *MdDAMb* was elevated in apical buds just before budbreak, resembling the expression patterns of peach *DAM5* and *DAM6*, which were regulated by cold exposure and inversely correlated with budbreak rate (Li et al., 2009; Jiménez et al., 2010; Leida et al., 2012b). Similarly, both *MdSVPa* and *MdSVPb* accumulated in apple buds during winter, reaching their peak during the coldest time of the year, followed by a transient decline before budbreak. At this stage, it is unclear if any of the apple *DAM* and *SVP* genes directly responds to cold exposure or if their expression reflects developmental events in the bud. Direct binding of cold response C-repeat binding factors (CBF) to C-repeat (CRT)/dehydration-responsive elements (DRE) in the promoter regions of apple *DAM* and *SVP* genes has been proposed (Wisniewski et al., 2015) and CBF-mediated enhanced DAM expression was also recently demonstrated in pear (Niu et al., 2016). CRT/DRE motifs have been identified upstream of the coding regions of *MdDAMa*, *MdDAMc* and *MdSVPb* (Mimida et al., 2015; Wisniewski et al., 2015), at least two CRT/DRE motifs (A/GCCGAC) are located within 2 kb upstream of *MdSVPa* transcription start site and a putative CRT/DRE motif has been identified in the upstream region of *MdDAMb*, but requires confirmation because of the poor sequence quality in that genomic region. The significance of the CBF pathway and the cross talk between cold and dehydration signaling (Shinozaki and Yamaguchi-Shinozaki, 2000) could therefore be of key importance in convergence of signals that regulate *DAM*/*SVP* expression and distinct aspects of dormancy and growth, but the molecular mechanisms underlying distinct expression of *DAM* and *SVP* genes in apple remain to be determined.

In *Arabidopsis*, *SVP* functions as a repressor of the floral transition during the vegetative phase and later it contributes to the specification of floral meristems, acting to supress floral organ development (Gregis et al., 2009). Hence, removal of *SVP* is necessary to enable flower organ development, consistent with general absence of apple *DAM* and *SVP* transcripts in mature flower and fruit tissues. To address their potential roles in early apple flower development, the predictable biennial bearing pattern of ‘Sciros’ was utilized (Bertelsen and Tustin, 2002). Low flowering and reduced cropping in ‘Off’ years results from the lack of floral induction on fruit-bearing trees in ‘On’ years and is accompanied by differential expression of several flowering regulator genes (Guitton et al., 2016). *MdDAM* genes in this study showed similar patterns regardless of fruit load, suggesting that *DAM* gene expression was not principally affected by the physiological states of apical buds. *MdSVPa* and *MdSVPb* were upregulated in buds of ‘Off’ trees in February, coinciding with the predicted prevalence of floral meristems in buds of non-bearing trees between 100 and 141 days after full bloom (DAFB) (Foster et al., 2003), indicating potential roles of *MdSVPa* and/or *MdSVPb* in floral meristem identity similar to *Arabidopsis* (Gregis et al., 2009). However, these roles may be minor, considering the normal flower development recorded in transgenic apple lines.

For the functional study, we successfully generated transgenic lines overexpressing a *DAM* and an *SVP* gene from apple. The low regeneration efficiency was consistent with previous reports (Sasaki et al., 2011; Wu et al., 2017), suggesting that overexpression of *SVP*-like genes strongly inhibits outgrowth of shoots in tissue culture, which may explain the limited number of reported transgenic approaches to functionally characterize *SVP/DAM* genes in woody perennial species. To our knowledge, this is the first report of homologous overexpression of *SVP*-like genes in a horticultural tree. The role in growth inhibition was corroborated by constrained axillary shoot growth and significantly delayed spring budbreak in transgenic *MdDAMb* and *MdSVPa* lines. The normal timing of leaf senescence, growth cessation and apical bud set suggested against a role in establishment of dormancy and the normal flower and fruit development suggested no or perhaps only minor roles in floral meristem and floral organ development. Previous studies demonstrated a general growth inhibitory function of *DAM* and *SVP* genes, but also a role in dormancy onset. Overexpression of Japanese apricot *DAM6* in transgenic poplar resulted in premature growth cessation and terminal bud set in autumn and delayed shoot outgrowth in spring (Sasaki et al., 2011). However, in kiwifruit, *SVP2* performed as a growth repressor once dormancy has been established, but appeared insufficient to suppress growth in permissive conditions (Wu et al., 2017). The current study suggests that *MdDAMb* and *MdSVPa* genes play similar growth inhibition roles in apple, without the capacity to promote growth cessation, consistent with their seasonal expression patterns. Therefore, *MdSVPa* and *MdDAMb* may maintain growth suppression upon establishment of dormancy, whilst *MdDAMa* and *MdDAMc* remain as potential candidates for regulation of growth cessation in autumn.

In summary, apple *DAM* and *SVP*-like genes likely play key roles in regulating growth and dormancy cycles in apple. This knowledge can help develop new varieties with budbreak and flowering dates which ensure optimal growth and adequate production in different growing regions and environments. The transgenic lines generated over the course of this study will help elucidate the genes, pathways and biochemical aspects underlying the growth inhibitory functions of *MdDAMb* and *MdSVPa*.

## Author Contributions

RW designed and performed experiments and prepared the manuscript. ST and SK provided technical assistance to RW. SDT designed the study of biennial bearing apple. RH and AA provided bioinformatics expertise and intellectual input. RM revised the manuscript. EV-G was involved in design of the study and planning the content, editing, and finalizing the manuscript. All authors read and approved the manuscript.

## Conflict of Interest Statement

The authors declare that the research was conducted in the absence of any commercial or financial relationships that could be construed as a potential conflict of interest. The reviewers JPL, TA and handling Editor declared their shared affiliation, and the handling Editor states that the process nevertheless met the standards of a fair and objective review.

## Supplementary Material

FIGURE S1 **Relative expression of *MdDAM* and *MdSVP* genes in apple apical buds during the season. (A)** *MdDAMb* expression during winter dormancy and active growth in spring. **(B)** *MdDAMb* expression from full bloom to leaf drop. The level of expression was normalized to three previously characterized apple reference genes MDP0000173025, MDP0000326399 and *MdActin* (GenBank accession number CN938023) (Espley et al., 2007; Bowen et al., 2014) and compared to reference gene *SGL29L* used in this study (**Figure 3**). Data points represent the mean ± SE of two biological replicates.
