# Supplementary figures and images for: SVP-like MADS Box Genes Control Dormancy and Budbreak in Apple
Source: Front Plant Sci. 2017 Apr 4;8:477. doi: 10.3389/fpls.2017.00477 (PMC5378812; doi:10.3389/fpls.2017.00477)

Supplementary Fig. 1

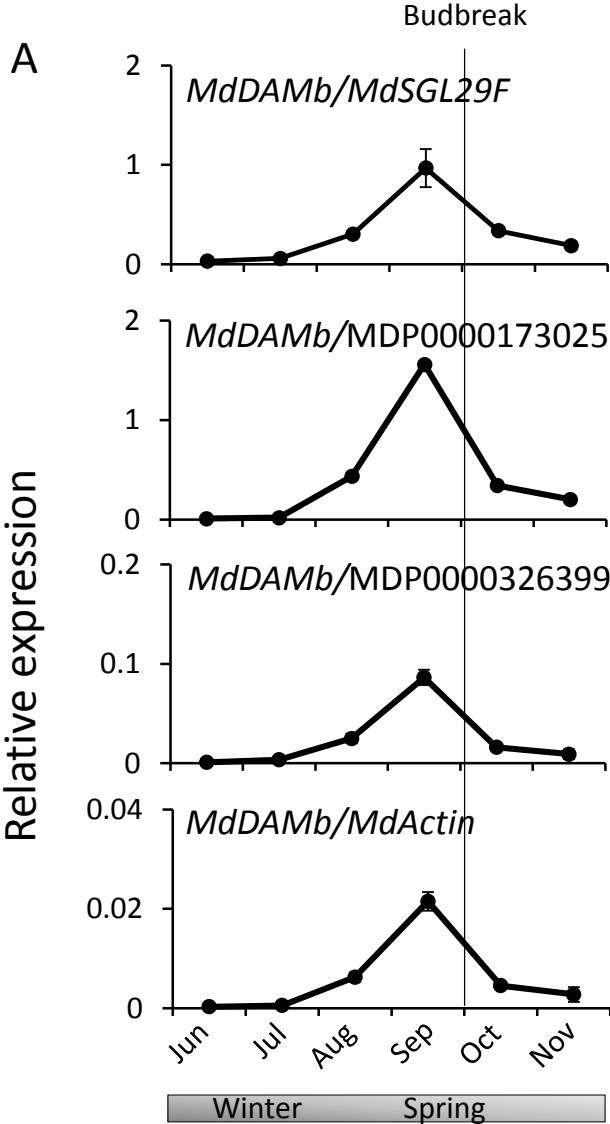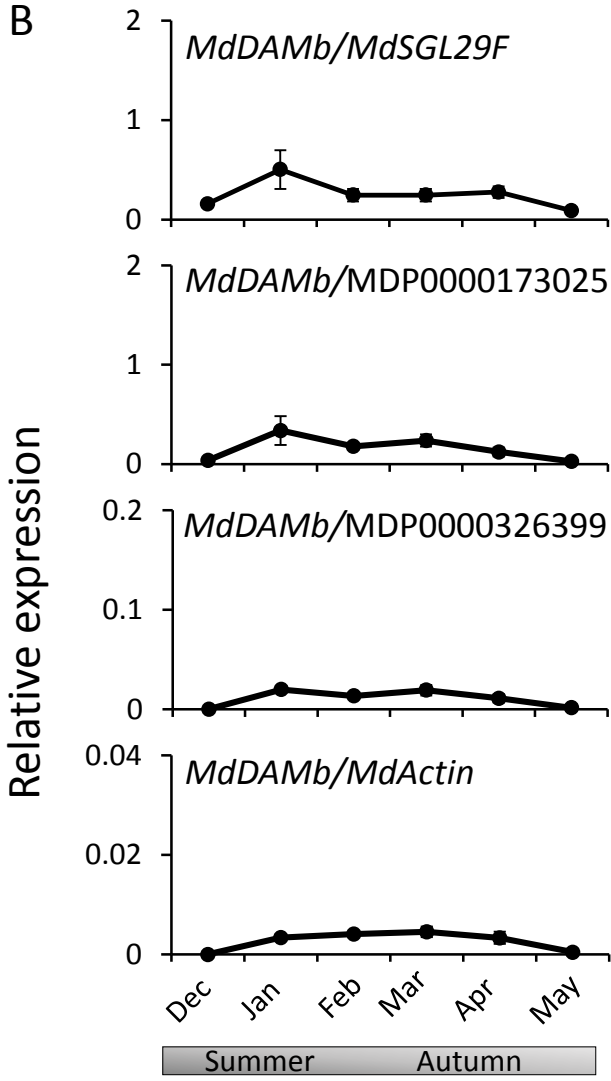

Supplement: FIGURE S1 — Relative expression of MdDAM and MdSVP genes in apple apical buds during the season. (A) MdDAMb expression during winter dormancy and active growth in spring. (B) MdDAMb expression from full bloom to leaf drop. The level of expression was normalized to three previously characterized apple reference genes MDP0000173025, MDP0000326399 and MdActin (GenBank accession number CN938023) (Espley et al., 2007; Bowen et al., 2014) and compared to reference gene SGL29L used in this study (Figure 3). Data points represent the mean ± SE of two biological replicates. [file Image_1.PDF]
